# Retrospective Evaluation of Lung Adenocarcinoma Patients Progressing on 1st Line Chemotherapy

**DOI:** 10.3390/medicina55110743

**Published:** 2019-11-16

**Authors:** Heikki Vilhonen, Samu Kurki, Tarja Laitinen, Samuli Hirsjärvi

**Affiliations:** 1 Division of Medicine, Department of Pulmonary Diseases, Turku University Hospital, 20521 Turku, Finland; 2 Department of Pulmonary Diseases and Clinical Allergology, University of Turku, 20521 Turku, Finland; 3 Auria Biobank, University of Turku, and Turku University Hospital, 20521 Turku, Finland; 4 Tampere University Hospital, 33521 Tampere, Finland; 5 Boehringer Ingelheim Finland Ky, 00180 Helsinki, Finland

**Keywords:** non-small cell lung cancer, adenocarcinoma, chemotherapy, real world data

## Abstract

*Background and Objectives:* Evaluation of data from electronic health care records could help in guiding towards more rational drug treatments. This single center study evaluated clinical characteristics that could be associated with disease progression. *Methods:* This was a real world data (RWD) study using existing data from the registries of a university hospital. Patients had lung adenocarcinoma and they had received 1st line treatment. Treatment patterns and survival parameters were characterized and clinical characteristics of the patients were evaluated together with their association with disease progression. *Results:* 80 stage III/IV patients fulfilling inclusion criteria were identified. Mean age was 62 years and 61% were men. In total, 65% were current smokers and 82% had performance status (ECOG) 0/1. Median progression free survival (mPFS) and median overall survival (mOS) for stage III and IV patients were 8.5 and 5.4 months, and 21.9 and 8.6 months, respectively. The study found that 69% of patients progressed within 9 months from the start of the 1st line treatment. Poor performance status (ECOG 3), male gender, and smoking suggested faster disease progression. Most had received cis/carboplatin-based treatment in the 1st line. Cisplatin regimens were associated with more complete responses and better PFS and OS than the carboplatin ones. *Conclusions:* By combining algorithmic and manual validation of electronic health care records, clinically valid characteristics and outcomes could be evaluated and presented. This approach forms a basis for tools such as quality registries that can guide treatment decisions.

## 1. Introduction

Prognosis for the majority of patients with advanced stage lung cancer has not changed significantly in the past decade. With an overall 5-year survival rate of 9–13%, the treatment of non-small cell lung cancer (NSCLC) remains a major clinical challenge [1]. For about 80–85% of patients with adenocarcinoma, there are currently no authorized options available for targeted treatment of driver mutations (such as EGFR, ALK) [2,3]. Therefore, there is a need for new treatment options. For example, the median overall survival (mOS) time that can be achieved with 2nd line therapy with traditional drugs such as docetaxel, pemetrexed, or erlotinib is 7–9 months [4,5,6]. On the other hand, recently approved immunotherapies (nivolumab, pembrolizumab, atezolizumab) can extend OS of selected patients up to nearly 20 months, as well as OS of unselected patients to about 14 months [7,8,9]. Recent studies also show promising results for pembrolizumab in the 1st line treatment in combination with a platinum doublet [10]. However, in order to remain rational and cost-effective, selection criteria of these patients in clinical practice still needs to be determined.

LUME-Lung 1 study evaluated the efficacy and safety of nintedanib in combination with docetaxel in patients with locally advanced/metastatic or recurrent NSCLC following failure of 1st line chemotherapy versus placebo plus docetaxel [11]. OS of patients with adenocarcinoma was prolonged by combination therapy of nintedanib + docetaxel versus docetaxel monotherapy from 10.3 to 12.6 months (HR (hazard ratio) 0.83; *p* = 0.04). In addition, it was shown in a pre-specified analysis that patients with progression during or shortly after 1st line therapy (within 9 months) benefited with an OS prolongation from 7.9 to 10.9 months (HR 0.75; *p* = 0.007). A further exploratory evaluation was performed for patients who progressed directly on 1st line therapy. In these patients, OS was also significantly prolonged from 6.3 to 9.8 months (HR 0.62; *p* = 0.03). In a recent analysis of European adenocarcinoma patients of LUME-Lung 1 study, an OS benefit of nearly five months was observed [12].

In contrast to driver mutations (EGFR, ALK, ROS1) and immunotherapies, at present there are no approved predictive diagnostics guiding the usage of anti-angiogenic therapies—such as nintedanib—although several drugs are available in this class [13]. Therefore, studies are ongoing to identify suitable bio-tumor markers that allow identification and selection of a more targeted population of patients most likely to benefit from the treatment. The LUME-Lung 1 study results proposed that one such population could be patients whose disease progresses immediately or shortly after the initiation of 1st line treatment.

The aim of this real world data (RWD) study was to evaluate the outcome of the treatments and clinical characteristics that are associated with disease progression of lung adenocarcinoma patients during and after the 1st line chemotherapy. In addition, an objective was to analyze and put into perspective quality of this RWD by algorithmic processing and manual screening. Such an approach could help in improving data recording in clinical practice, which leads to coherent register data and tools such as quality registries.

## 2. Materials and Methods

### 2.1. Patient Selection

The initial patient cohort was identified from the pathology registry of the Turku University Hospital for years 2004–2013 (catchment population of ~500,000). All patients who had histologically verified (lung/bronchial biopsy or surgical biopsy) malignant neoplasm of lung or bronchus (tissue SNOMED (systematized nomenclature of medicine) T26-T28) were included. Patients whose diagnosis was based only on cytology or biopsy from metastasis were excluded. Cytological samples were not included in the register combining samples and clinical information. Using the patients’ electronic medical records, we gathered the data on their clinical diagnosis, reports on their inpatient and outpatient events, clinical procedures, hospital pharmacy records, radiation therapy, laboratory and imaging results, and the date of death.

**Figure 1:**
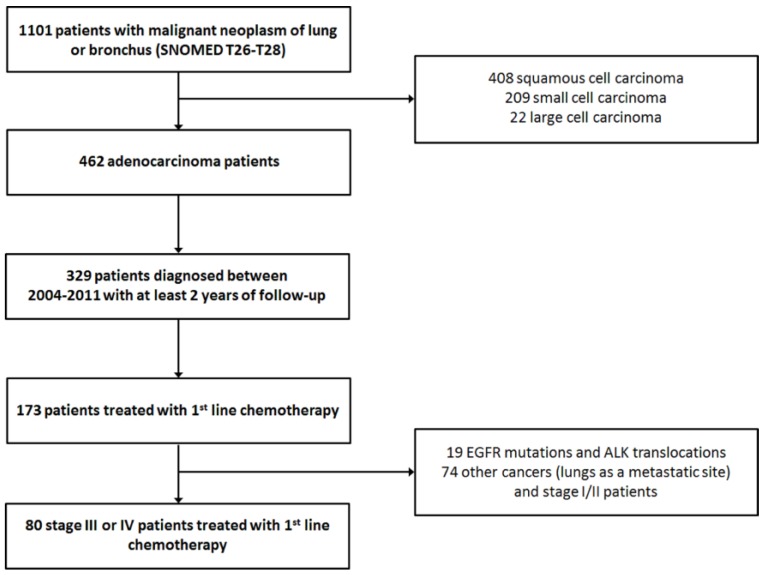
Patient selection for the analyses.

First step of filtering the identified patient cohort was to identify the patients with lung adenocarcinoma. Only patients who were diagnosed before the year 2011 were included; thus, every patient could be followed for two years or until death (Figure 1). These patients were then cross-referenced with the hospital registry on chemotherapy regimens.

To verify the diagnosis and stage of the disease, given chemotherapy combinations and treatment responses, corresponding clinical patient records were analyzed manually by a clinician. Progression was determined from radiological, pathological and clinical records. In unclear cases, the clinician assessed the radiology images retrospectively using the RECIST (response evaluation criteria in solid tumors) criteria. For statistical analysis, these digitally and manually curated data tables were combined into a single one.

Clinical variables used in the analysis were observed at the beginning of 1st line treatment: age (numerical), gender (female/male), stage (3/4), previous surgery (no/yes), radiation therapy for the primary cancer site (no/yes, radical/palliative), smoking (never/current/quit), and Eastern Cooperative Oncology Group (ECOG) performance status (numerical). A variety of baseline laboratory values were evaluated (Table S1). The test results that were available for >90% of the patients within three months before 1st line treatment, were included to the analysis. Descriptive neutrophil/leukocyte (neutr/leuk) and thrombocyte/leukocyte (trom/leuk) ratios were calculated from the laboratory values. Two survival parameters were defined for this patient cohort, estimated from the beginning of 1st line chemotherapy: overall survival (OS) and progression free survival (PFS). Progression moment was defined as a mention of disease progression in patient records, i.e., radiological progression. Response was evaluated by imaging every 2–3 cycles during active treatment, every 3–6 months during follow up or with the advent of new symptoms.

Patient years and use of hospital resources were calculated from the beginning of the 1st line treatment until death or end of follow-up. Hospital resources included outpatient visits, inpatient periods, inpatient days, and laboratory visits.

### 2.2. Statistical Methods

This study was based on an evaluation of retrospective samples and clinical data and therefore descriptive statistics were mostly used to describe the clinical/biological characteristics of the patient population. Patient outcomes, such as OS or PFS, were analyzed using univariate Kaplan-Meier survival analysis or multivariate Cox regression to adjust for potential covariates. Chi-squared test was used for discrete categories; p values and 95% confidence intervals were calculated where applicable.

The study was based on existing data and no interventions were performed. The pseudonymized research data was analyzed in Turku University Hospital in-house data analysis platform to maximize the privacy of the study subjects. The analysis platform enforces two-level authentication and logging, and the data set was archived for later reference. The study was conducted in accordance with the ethical principles that have their origin in the current Declaration of Helsinki and were consistent with Good Epidemiology Practices, code of standards and ethics for survey research, and other applicable local laws and regulatory requirements. This registry-based study design was approved by the Office of the Data Protection Ombudsman, Finland (20 October 2016, approval code AB16-9882), and the data gathering and analysis was performed with the permission of Turku University Hospital.

## 3. Results

In total, 173 lung adenocarcinoma patients were identified who had received at least 1st line chemotherapy (Figure 1). During the clinical re-evaluation, a number of cases turned out not to be primary lung cancers. Also stage I/II patients and patients with known EGFR mutation or ALK translocation were excluded. As expected, a greater proportion of adenocarcinoma patients was men (61%) and older than women (Table 1). Only 20 (25%) of these late stage patients had surgical sample in the register; 15 (19%) had received definitive radiotherapy for their disease. Most of these were stage III patients: 16 with surgical sample and 14 with radiotherapy. In total, 16 patients with surgical sample had received (adjuvant) chemotherapy less than 3 months after the surgery. Chemotherapy and radiotherapy were not concurrent for any of the patients. Data extraction from patient records revealed that 65% were current smokers at the time of diagnosis. Most had a good performance status with ECOG 0/1 representing 82% of the patients. As the evaluation of performance status was performed retrospectively, some patients with ECOG 3 had also received chemotherapy. These patients were disabled by reasons that where not contradictory with drug treatment.

The most common regimens administered to patients were cis/carboplatin combined with gemcitabine (*n* = 33), followed by platinum doublets containing pemetrexed (*n* = 19) and vinorelbine (*n* = 8) (Table 2). Pemetrexed was the most used single agent (*n* = 5). Cisplatin based regimens resulted in more complete responses (CR) than the carboplatin ones (14.7% vs. 3.2%). Patients treated with carboplatin doublets had slightly more partial responses (PR) and stable disease (SD) but, on the other hand, more disease progression (PD) as a response. Single agents and other combinations were not frequently used, resulting more often in stable disease or disease progression.

**Figure 2:**
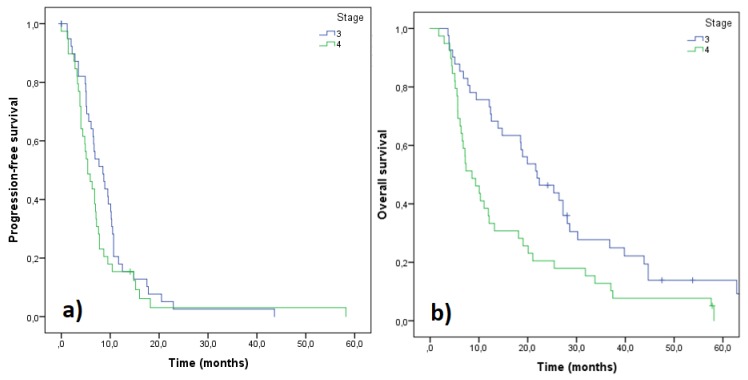
Progression free survival (PFS) (**a**) and overall survival (OS) (**b**) of the patients based on their staging. Stage III (*n* = 41), stage IV (*n* = 39).

**Figure 3:**
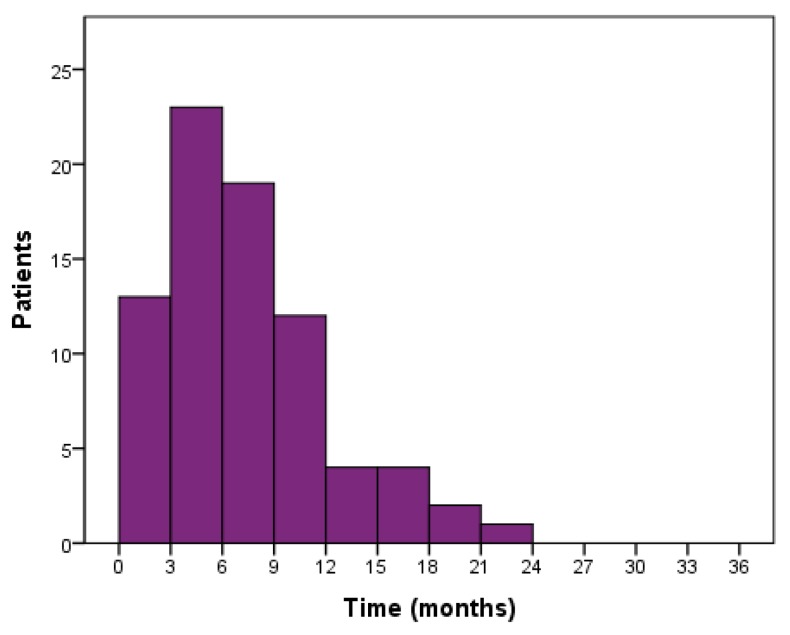
PFS distribution of 1st line treated patients (*n* = 80).

Automated algorithm using the start of 2nd line treatment or death as an indicator of PFS end-date provided median PFS of 11.2 months for stage III and 7.2 months for stage IV patients. Manually curating the data using radiologically and clinically determined date of progression produced median PFS of 8.5 and 5.4 months for stages III and IV, respectively (Figure 2). Median OS for stage III and IV patients were 21.9 and 8.6 months, respectively (Figure 2). Median PFS for all 80 patients was 6.7 months while 55 patients (68.8%) progressed within 9 months (Figure 3). Median PFS for patients who were able to start 2nd line treatment (*n* = 35) was 9.5 months and 16 of those patients (45.7%) had progressed within 9 months.

ECOG score 3 reduced significantly PFS in the cohort of 80 patients. Male gender was predictive for fast progression without reaching statistical significance (Table 3). On the other hand, patients with prior surgical operation and radical radiotherapy had better prognosis.

In addition, disease progression (before or after 9 months) and age (under or over 60 years) were used as discrete categories to compare variables. Average ages of patients progressing within and after 9 months were the same: 61.9 years (*n* = 35) and 61.3 years (*n* = 45), respectively. There was a statistically significant prevalence of men in the <9 months group, 74.3%. Stage IV patients progressed clearly faster than stage III patients (68.6% in the <9 group) as well as patients without a surgical intervention in the records (94.3% in the <9 group). Only 1 patient had received radiotherapy in the faster progressing <9 group. Current smokers progressed faster than never smokers or also ex-smokers. Of the slower progressing patients (>9 months), 90.9% had ECOG 0-1. Thus, these slower progressing patients were in better physical condition than the faster progressing patients: 81.3% of the patients in the <9 months group had ECOG 1-3. This comparison based on the disease progression speed is summarized in Table 4. The other discrete comparison divided patients in <60 years and ≥ 60 years groups (Table S5). Average ages of patients in the former and latter groups were 54.4 (*n* = 32) and 66.3 (*n* = 48) years, respectively. No statistical significances were found in this comparison based on age. More men were in the younger <60 years group. Current smokers were more common in the younger group compared to the older subgroup. Stage III patients were younger than stage IV patients.

No baseline laboratory value was linked to progression of the disease according to the continuous PFS variable analysis (Table S2). In the discrete variable comparison, baseline MCH (mean corpuscular hemoglobin), MCV (mean corpuscular volume), and CRP (C-reactive protein) were significantly higher in the >9 months group, and CRP in the <60 years group (Supplementary Material, Tables S3–S4). Nevertheless, differences in MCH and MCV were within the common reference margins. Neutr/leuk higher than median (0.65) was associated with decreased PFS (*p* = 0.013). Inversely, higher than median (>38) trom/leuk ratio was associated with longer PFS (*p* = 0.018).

**Figure 4:**
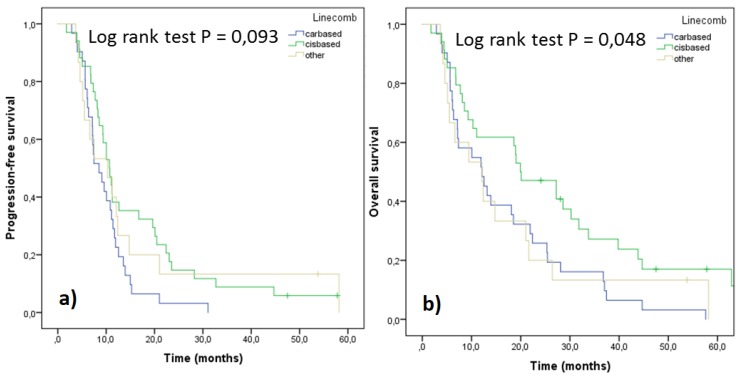
PFS (**a**) and OS (**b**) of the patients according to their 1st line treatment (n = 80).

**Figure 5:**
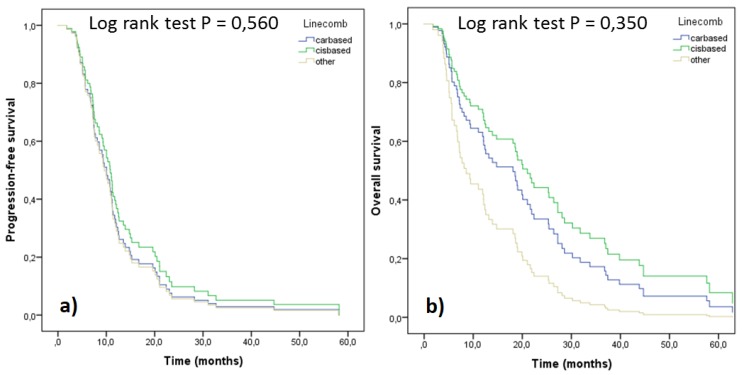
PFS (**a**) and OS (**b**) of the patients according to their 1st line treatment, adjusted for stage and performance status (*n* = 80).

Survival parameters PFS and OS were analyzed as a function of cis/carboplatin doublets and other drugs administered (Figure 4). Significantly better survival in terms of both parameters was observed with cisplatin regimens compared to carboplatin ones. However, when the treatment outcomes were adjusted for stage and performance status, no significant differences were seen, although the trends still favored cisplatin doublets (Figure 5). In order to assess differences between pemetrexed and gemcitabine, outcomes of these cisplatin doublets were compared (cisplatin-pemetrexed vs. cisplatin-gemcitabine). No differences were observed in PFS (*p* = 0.832) and OS (*p* = 0.813).

Use of hospital resources was evaluated according to the disease progression (before or after nine months) and presented per patient years. Use of each selected resource per patient year was greater in the faster progressing group (<9 months group vs. >9 months group): outpatient visits 35.4 vs. 21.8; inpatient periods 6.8 vs. 2.2; inpatient days 41.3 vs. 10.7; and laboratory visits 56.6 vs. 24.3.

## 4. Discussion

There is an increasing need for RWD. These data can be used to guide patient selection towards more specific and rational drug treatments, as well as to support authorities in drug reimbursement decisions. Treatment outcome is evident for these decisions, but other data such as the use of hospital resources can be useful as well. As we showed, use of hospital resources was greater if the disease progressed faster. Disease progression and, thus, costs could be possibly reduced by drug treatments. Therefore, this kind of quality registries based on RWD play an important role in monitoring disease treatment and healthcare outcome [14]. Standardized data collection methods, a topic that we also assessed in this study, are a cornerstone in quality registries. Moreover, evidence based on RWD can fill information gaps created by traditional clinical trials and their strict patient selection criteria. Emergence of clinical biobanks that offer structured register data is a solution to address the need of supportive evidence. Finnish biobank ecosystem offers a straightforward and compliant access to these registries, and facilitates elaboration of further studies [15].

Also, there is a growing interest to understand patient profiles based on the disease progression characteristics. For example, a subset of patients has shown to progress fast upon immunotherapy [16]. As an anti-angiogenic drug, nintedanib has shown to benefit patients who had progressed fast on 1st line treatment [11]. Therefore, the aim was to search for clinical characteristics that could predict the course of the disease during and after the 1st line treatment.

The amount of 1st line treated patients (*n* = 80) was small compared to the whole adenocarcinoma population in the register (*n* = 462). Of all 1st line treated patients (*n* = 173), a large proportion were cancers of which lungs were a metastatic site. These were excluded from the analysis. In total, 80 cases were confirmed to be stage III or IV lung cancer patients that had received 1st line chemotherapy (19 were excluded because of EGFR/ALK positivity). This revealed a limitation in the register data: ICD-10 and similar codes differentiate primary lung cancer and lungs, as a metastatic site, but there might be imperfections in documenting diagnosis in registries. However, this register data was epidemiologically inclusive as it covered all cancer patients of the region. According to the Finnish Biobank Act, archive data from all patients could be extracted until 2013. After that, the procedure was modified to require individual patient consent for study purposes. Therefore, it could be assumed that the selected observation period (2004–2013) provided a comprehensive patient population. Taking all of this into account, our 1st line treated cohort approached to the proportions (per all adenocarcinoma patients) observed usually in the real life setting during a similar observation period (period starting from mid-2000 or earlier) [17]. Surgery was performed to 20 patients of the 80. The majority of these were stage III patients (patients with a surgical sample are probably well represented in the register). Obviously, according to the clinical practices, operated patients were generally in better condition or the disease was more local. In these cases, the benefit of the surgery was greater. Nevertheless, stage III and IV patients were both included in this study. In our opinion, this selection described the situation better in a real-life setting and reflected the study objective: all these patients had received drug treatment for their metastatic or recurrent disease, and, progressed during or after that treatment.

PFS and OS outcome revealed the difference between stage III and IV patients. These data corresponded to other findings from real world sources (adenocarcinoma/non-squamous histologies). For example, Peters et al. [18] reported mOS of 416 days (~14 months) and 276 days (~9 months) for stage IIIB and IV patients, respectively. Abernethy et al. [16] observed mOS 10.0 months for stage IV patients, and Nadler et al. [19] mOS 12.3 months for all 1st line treated patients (including targeted treatments). As a difference, our stage III patients, with mOS 21.9 months, included the adjuvant treated patients. Our mOS for stage IV patients (8.6 months) was defined as from the start of 1st line treatment until death (opposite to: from diagnosis to death). Reported mPFS of 10 months and mOS of 20 months for stage III patients (all histololgies) [20] were also similar to our results. When compared to clinical studies of platinum doublets, PFS in this study was similar: 6.7 months (vs. ~5 months [21]). In total, 35 of the 80 1st line treated patients (43.8%) obtained further 2nd line treatment. This was well in line with other real-world findings [17,18]. Manual data screening revealed that algorithmic validation of electronic health care records was not precise in defining the progression moment to be used as PFS. These observations are important when considering further application of RWD.

As a consequence, 9 months was chosen as discrete when assessing effect of variables on disease progression because it was close to the average PFS of this cohort. Considering nintedanib, OS data from its LUME-Lung 1 study described the time since start of 1st line therapy as a predictive marker for extended survival in 2nd line patients. The cut-off of 9 months was established, as this represented the moment where the hazard ratio point estimate was <1 and for which the width of the 95% CI was small [22]. Therefore, the 9 months discrete chosen for this register study turned out to be an appropriate divider. A major proportion of the patients, 68.8%, progressed within 9 months from the 1st line start. Although the results revealed that these faster progressing patients were in worse physical condition, 45.7% of the patients who were able to receive 2nd line chemotherapy belonged to the <9 months group. Therefore, an essential fast progressing patient pool which is eligible for 2nd line treatment—such as nintedanib—exists in clinical practice.

Finally, only clinical variables that seemed to predict faster disease progression were male gender, poor ECOG score, smoking, and elevated CRP at baseline. Impaired performance status has been linked to disease progression also in other studies [19]. In addition, performance status is a robust characteristic that can act as surrogate to comorbidities when predicting outcome [23]. Radiation treatment resulted in slower disease progression because only radical radiation treatment was selected as a variable (no palliative radiation). Same applied to surgery: most of the available surgical samples were from radically operated patients at earlier stage of their disease, with only few palliatively operated specimens in the register.

Neutrophil-to-lymphocyte (leukocyte) ratio (NLR) and platelet (thrombocyte)-to-lymphocyte ratio (PLR) have been reported to be prognostic for poor outcome in cancer including NSCLC [24]. Immunotherapies, nivolumab as an example, work the best the longer the time from completion of 1st line regimen to treatment initiation [7]. Similarly, higher baseline NLR and PLR were found to be associated with poorer response to nivolumab [25]. Therefore, elevated inflammation related parameters NLR and PLR could guide treatment decisions with agents—such as nintedanib—that provide better response in fast progressing disease. In our data, this kind of tendency was only seen in the case of NLR: higher ratio at the baseline seemed to result in faster disease progression.

Today, the majority of electronic health record data are narrative text. Efforts are made to structure text using natural language procession. A Python 3.5 based universally applicable tool for mining of clinical text has been developed [26]. The tool has been previously used to collect information about various parameters such as medications, metastasis status, ejection fraction, and proliferation index. Smoking status, as presented in the results of this study, was extracted by this tool. To assess correctness of the results, an accuracy of 83% was achieved when comparing the smoking status by the algorithm with manual (human) data extraction. This showed that automated data extraction is a powerful tool for register studies. In addition, this kind of approach helps to improve data registration practices which, in the end, lead to better treatment of patients.

The long time span of this study reflected changes both in diagnostic and in approach to lung cancer treatment during this period. One such change, revealed by the current study, was the use of subsequent approach to chemotherapy and radiotherapy in the patient cohort (opposite to the current concurrent approach). Until now, platinum doublets have been the standard of care in the 1st line treatment of NSCLC. In this study, gemcitabine was the preferred combination partner for platinum because the register contained data also from patients who were treated before the adoption of pemetrexed (2004 onwards). Cisplatin combinations resulted in better PFS and OS compared to carboplatin combinations with PFS being significantly better. Cisplatin is, indeed, considered to be more effective, although more toxic than carboplatin [27]. Therefore, the survival parameters were also adjusted for stage and performance status: in this analysis, the differences were not that substantial anymore. However, cisplatin doublets provided more complete and partial responses compared to carboplatin doublets. Similarly, disease progression was more often a response to a carboplatin-based treatment. For NSCLC patients of non-squamous histology, PFS and OS for cisplatin-gemcitabine or cisplatin-pemetrexed combinations are approximately five and 10 months, respectively, with five cycles administered being the median [21]. According to our data from clinical practice, PFS and OS values were similar. Treatment length in this study (median duration between 60 and 90 days) was similar to other RWD: Peters et al. [18] reported that 49% of patients received less than four cycles (in 28 days) of 1st line treatment. In this study, the length was calculated from the beginning of the 1st cycle until the administration of the last cycle (not the cycle end), which might have “shortened” the observed treatment length. If observed separately, vinorelbine stood out with its superior survival results compared to the other drugs. It is commonly used as adjuvant therapy after surgery and as a part of chemoradiotherapy [28]. Also, in this study, it was probably administered to the stage III patients who were in earlier phases of their treatments and, therefore, the outcome followed the known results from literature and experiences from the clinic.

## 5. Conclusions

Data from electronic health records are excellent sources to generate a hypothesis for subsequent, prospective studies. Likewise, it offers a straightforward manner to assess validity of hypothesis and clinical trial results in clinical practice. For example, in this study, the efficacy outcome of drug treatments was consistent with known clinical trial results. Similarly, the variables that were identified to suggest faster disease progression—poor performance status, male gender, and smoking—could be stratified in further, prospective trials. In addition to identifying those clinical characteristics, this study clearly helped in developing RWD methodology and related practices in a local level. By combining algorithmic and manual validation, clinically valid characteristics and outcomes could be evaluated and presented. Examples of this included the accurate determination of progression free time and extraction of smoking status. Methodology that we presented could form the basis for tools such as quality registries that can guide treatment decisions.

## Figures and Tables

**Table 1:** Baseline characteristics of the analyzed lung adeno carcinoma patients (*n* = 80).

Age		61.6 ± 8.0
Gender	Female	31 (39%)
	Male	49 (61%)
Stage	III	41 (51%)
	IV	39 (49%)
Previous treatments ^1^	Surgery	20 (25%)
	Definitive radiotherapy	15 (19%)
Smoking	Never	11 (14%)
	Current	52 (65%)
	Quit	17 (21%)
ECOG ^2^*	0	23 (29%)
	1	42 (53%)
	2	6 (8%)
	3	5 (6)

^1^ Only surgery or radiotherapy with curative intent before or as a part of 1st line therapy is listed. ^2^ Information of 4 patients missing. * Eastern Cooperative Oncology Group performance status.

**Table 2:** Chemotherapies and treatment responses at end of the 1st line treatment.

		Treatment Response, n (%)				
Drug	*n*	CR	PR	SD	PD	n/a
Cisplatin-gemcitabine	16	5 (14.7)	14 (41.2)	8 (23.5)	6 (17.6)	1 (2.9)
Cisplatin-pemetrexed	11					
Cisplatin-vinorelbine	6					
Cisplatin-gemcitabine-bevacizumab	1					
Carboplatin-gemcitabine	17	1 (3.2)	15 (48.4)	8 (25.8)	7 (22.6)	-
Carboplatin-pemetrexed	8					
Carboplatin-vinorelbine	2					
Carboplatin-etoposide	1					
Carboplatin-gemcitabine/vinorelbine	1					
Carboplatin-paclitaxel	1					
Carboplatin-paclitaxel/vinorelbine	1					
Pemetrexed	5	2 (13.3)	3 (20.0)	6 (40.0)	4 (26.7)	-
Platin-gemcitabine	3					
Platin-vinorelbine	2					
Gemcitabine	2					
Docetaxel-gemcitabine	1					
Gemcitabine/pemetrexed	1					
Vinorelbine	1					

CR = complete response, PR = partial response, SD = stable disease, PD = progressive disease.

**Table 3:** Hazard ratios (HR) with 95% confidence intervals (CI) of clinical variables explaining time to disease progression. Female gender, smoking status ‘never’ and ECOG 0 were reference categories.

Variable	HR	Lower 95% CI	Upper 95% CI
Age	0.970	0.937	1.003
Male gender	1.732 *	1.051	2.853
Stage (IV)	1.128	0.655	1.942
Surgery (yes)	0.438 *	0.242	0.795
Radiation (yes)	0.882	0.534	1.457
Smoking (current)	0.851	0.426	1.699
Smoking (quit)	1.244	0.590	2.622
ECOG (1)	1.170	0.665	2.058
ECOG (2)	2.373	0.915	6.155
ECOG (3)	3.049 *	1.052	8.836

* *p* <0.05 vs. reference category.

**Table 4:** Comparison of distribution of clinical variables using disease progression (>9 or <9 months) as discrete.

		PFS as Discrete		
		>9 Months, n (%)	<9 Months, n (%)	*p* Value
Gender	Female	22 (48.9)	9 (25.7)	0.040
	Male	23 (51.1)	26 (74.3)	
Stage	3	30 (66.7)	11 (31.4)	0.003
	4	15 (33.3)	24 (68.6)	
Surgery	No	27 (60.0)	33 (94.3)	0.001
	Yes	18 (40.0)	2 (5.7)	
Radiation treatment	No	31 (68.9)	34 (97.1)	0.001
	Yes	14 (31.1)	1 (2.9)	
Smoking status	Never	9 (20.0)	2 (5.7)	0.150
	Current	26 (57.8)	26 (74.3)	
	Quit	10 (22.2)	7 (20.0)	
ECOG	0	17 (38.6)	6 (18.8)	0.193
	1	23 (52.3)	19 (59.4)	
	2	2 (4.5)	4 (12.5)	
	3	2 (4.5)	3 (9.4)	

## Supplementary Materials

The supplementary tables are available online at https://www.mdpi.com/1010-660X/55/11/743/s1, Table S1: Baseline laboratory measurements, Table S2: Laboratory measurements. Cox regression to model PFS, Table S3: Laboratory measurements. Variable comparison, PFS as discrete (9 months), Table S4: Laboratory measurements. Variable comparison, age as discrete (60 years), Table S5: Comparison of distribution of clinical variables using age as discrete.
