# Supplementary material for: Retrospective Evaluation of Lung Adenocarcinoma Patients Progressing on 1st Line Chemotherapy
Source: Medicina (Kaunas). 2019 Nov 16;55(11):743. doi: 10.3390/medicina55110743 (PMC6915390; doi:10.3390/medicina55110743)
Supplement: Supplementary file 1 [file medicina-55-00743-s001.pdf]

Supplementary Material

Baseline laboratory measurements.

	Eryt	Hb	HKR	Leuk	Neut	Trom	MCH	MCV	AFOS	ALAT	CRP	Krea
Mean	4.40	132.03	0.40	8.90	5.92	329.15	30.20	90.18	92.51	23.27	30.48	66.85
SD	0.45	15.52	0.04	3.33	3.04	96.64	2.03	5.06	52.70	15.10	54.39	14.64
N	76	76	76	76	71	76	76	76	72	74	69	76

Laboratory measurements. Cox regression to model PFS.

	Eryt	Hb	HKR	Leuk	Neut	Trom	MCH	MCV	AFOS	ALAT	CRP	Krea
p value	0.098	0.663	0.210	0.318	0.186	0.433	0.843	0.135	0.593	0.299	0.055	0.541
HR	2848.66	0.95	0.00	1.56	0.52	1.00	0.90	1.49	1.00	1.01	1.01	1.01
Lower 95% CI	0.23	0.77	0.00	0.65	0.20	0.99	0.31	0.88	0.99	0.99	1.00	0.99
Upper 95% CI	3.55E07	1.18	1.69E16	3.71	1.37	1.01	2.60	2.52	1.01	1.04	1.02	1.03

Laboratory measurements. Variable comparison, PFS as discrete (9 months).

		Age	Eryt	Hb	HKR	Leuk	Neut	Trom	MCH	MCV	AFOS	ALAT	CRP	Krea
>9 m.	Mean	61.27	4.39	134.88	0.40	8.27	5.34	312.74	30.86	91.56	84.78	21.02	16.50	67.16
	SD	8.05	0.40	13.03	0.04	3.37	3.10	70.81	1.73	4.23	36.48	13.02	46.65	15.39
	N	45	43	43	43	43	41	43	43	43	40	42	38	43
≤9 m.	Mean	61.91	4.39	128.30	0.39	9.71	6.71	350.52	39.33	88.39	102.19	26.22	47.61	66.44
	SD	8.14	0.51	17.78	0.05	3.14	2.82	120.31	2.10	5.53	67.17	17.22	58.92	13.84
	N	35	33	33	33	33	30	33	33	33	32	32	31	33
	p value	-	0.997	0.066	0.161	0.062	0.060	0.091	0.001	0.006	0.165	0.144	0.017	0.833

Laboratory measurements. Variable comparison, age as discrete (60 years).

		Age	Eryt	Hb	HKR	Leuk	Neut	Trom	MCH	MCV	AFOS	ALAT	CRP	Krea
<60	Mean	54.42	4.40	131.68	0.40	9.26	6.18	326.71	30.04	90.21	83.31	20.00	52.46	65.11
	SD	5.84	0.44	14.97	0.04	3.94	3.51	95.28	2.06	5.17	24.83	9.05	80.75	15.21
	N	32	28	28	28	28	27	28	28	28	26	26	24	28
≥60	Mean	66.31	4.39	132.23	0.40	8.69	5.76	330.56	30.29	90.17	97.72	25.04	18.76	67.87
	SD	5.35	0.46	16.98	0.05	2.94	2.73	98.40	2.03	5.05	62.95	17.36	27.57	14.37
	N	48	48	48	48	48	44	48	48	48	46	48	45	48
	p value	-	0.924	0.833	0.998	0.473	0.571	0.868	0.600	0.969	0.268	0.172	0.013	0.432

Comparison of distribution of clinical variables using age as discrete.

		Age as discrete		
		<60 years, n (%)	≥60 years, n (%)	p value
Gender	Female	10 (31.3)	21 (43.8)	0.350
	Male	22 (68.8)	27 (56.3)	
Stage	3	18 (56.3)	23 (47.9)	0.501
	4	14 (43.8)	25 (52.1)	
Surgery	No	23 (71.9)	37 (77.1)	0.609
	Yes	9 (28.1)	11 (22.9)	
Radiation treatment	No	26 (81.3)	39 (81.3)	1.000
	Yes	6 (18.8)	9 (18.8)	
Smoking status	Never	2 (6.3)	9 (18.8)	0.111
	Current	25 (78.1)	27 (56.3)	
	Quit	5 (15.6)	12 (25.0)	
ECOG	0	10 (32.3)	13 (28.9)	0.662
	1	18 (58.1)	24 (53.3)	
	2	1 (3.2)	5 (11.1)	
	3	2 (6.5)	3 (6.7)	
